# Spot urinary microalbumin concentration, metabolic syndrome and type 2 diabetes: Tehran lipid and glucose study

**DOI:** 10.1186/s12902-022-00976-x

**Published:** 2022-03-08

**Authors:** Zahra Gaeini, Zahra Bahadoran, Parvin Mirmiran, Reza Norouzirad, Asghar Ghasemi, Fereidoun Azizi

**Affiliations:** 1 Nutrition and Endocrine Research Center, Research Institute for Endocrine Sciences, Shahid Beheshti University of Medical Sciences, No. 24, Sahid-Erabi St, Yemen St, Chamran Exp, P.O.Box: 19395-4763, Tehran, Iran; 2 Department of Biochemistry, School of Paramedical Sciences, Dezful University of Medical Sciences, Dezful, Iran; 3 Endocrine Physiology Research Center, Research Institute for Endocrine Sciences, Shahid Beheshti University of Medical Sciences, Tehran, Iran; 4 Endocrine Research Center, Research Institute for Endocrine Sciences, Shahid Beheshti University of Medical Sciences, Tehran, Iran

**Keywords:** Microalbumin, Urinary creatinine, Metabolic syndrome, Type 2 diabetes

## Abstract

**Aim:**

This study aimed to determine the association of urinary microalbumin concentrations with type 2 diabetes mellitus (T2DM), metabolic syndrome (MetS), and its phenotypes. The optimum cut-off values of urinary microalbumin and microalbumin-to-creatinine ratio (MCR) for predicting the chance of having T2DM and MetS were also defined.

**Methods:**

Adult men and women (*n* = 1192) participated in the sixth phase (2014-2017) of the Tehran Lipid and Glucose Study (TLGS), with completed data, were included in the analyses. Odds ratios (ORs) (and 95% confidence intervals (CIs)) of T2DM, MetS, and its components across tertile categories of urinary microalbumin concentrations were estimated using multivariable logistic regressions. The optimal cut-off points of urinary microalbumin and MCR were determined using the receiver operator characteristic (ROC) curve analysis.

**Results:**

Participants’ mean (±SD) age was 44.9 (±14.0) years, and 44.6% of the participants were men. The prevalence of microalbuminuria was 14.4%. Chance of having T2DM was significantly higher in the highest tertile of urinary microalbumin concentration (OR = 2.29, 95% CI = 1.43-3.67) and MCR (OR = 1.82, 95% CI = 1.15-2.89). Subjects with the highest urinary microalbumin concentration were more likely to have MetS (OR = 1.66, 95% CI = 1.17-2.35), hypertension (OR = 1.63, 95% CI = 1.16-2.30) and hyperglycemia (OR = 1.78, 95% CI = 1.24-2.56). No significant association was observed between urinary microalbumin concentrations and other components of MetS. The optimal cut-off points of urinary microalbumin for predicting the chance of having T2DM and MetS were 14.0 and 13.6 mg/L, respectively.

**Conclusions:**

Elevated spot urinary microalbumin, below the values defined as microalbuminuria, was associated with the chance of having T2DM and MetS.

**Supplementary Information:**

The online version contains supplementary material available at 10.1186/s12902-022-00976-x.

## Background

Microalbuminuria, defined as urinary albumin excretion of 20-200 mg/L [1], is an early marker of chronic kidney disease (CKD) and is also associated with the increased risk of cardiovascular disease, all-cause mortality, and metabolic disorders, including type 2 diabetes mellitus (T2DM) and metabolic syndrome (MetS) [2–5]. The prevalence of microalbuminuria among Asian, European and US population has been estimated about 5.0, 6.7 [6–8], and 5.0-7.8%, respectively [9].

A direct association has been reported between urinary microalbumin concentrations and metabolic disorders; epidemiologic evidence demonstrates that the prevalence of microalbuminuria is significantly higher in individuals with MetS [9–11] and T2DM [12, 13]. Non-diabetic individuals with abdominal obesity or dyslipidemia also had a higher risk of developing microalbuminuria [14, 15]. The associations between various components of MetS or T2DM and microalbuminuria, to some extent, were conflicting [10, 11, 16, 17].

The cut-off values of urinary microalbumin have primarily been defined for proteinuria; no study in the general population has addressed the diagnostic efficacy of random-spot urinary microalbumin concentration for predicting the risk of T2DM and MetS. Considering the increasing prevalence of renal dysfunction, the determination of population-specific and outcome-specific cut-off values of urinary microalbumin for predicting metabolic disorders seem to be essential. Therefore, this cross-sectional study aimed to evaluate the association between urinary microalbumin concentration and microalbumin-to-creatinine ratio (MCR) with the chance of having T2DM, MetS, and its phenotypes among Iranian adults. We also defined the optimum cut-off values of urinary microalbumin concentration and MCR to predict the chance of T2DM and MetS in our population.

## Methods

### Study population and measurements

This study was conducted within the framework of a population-based study, the Tehran Lipid and Glucose Study (TLGS), an ongoing prospective study initially started in 1999 to investigate and prevent non-communicable diseases among the Tehranian population [18]. Adults men and women (*n* = 1192, age ≥ 19 years) with complete data on fasting spot urinary concentrations of microalbumin, sodium (Na), potassium (K), creatinine (Cr), as well as demographics, anthropometric, and other biochemical measurements in the sixth examination of the TLGS (2014-2017) were included in this study. Trained interviewers of TLGS collected anthropometric measurements, including participants’ weight, height, and waist circumference (WC). Detailed information has been described elsewhere [18].

Systolic (SBP) and diastolic (DBP) blood pressures were measured using a standard mercury sphygmomanometer calibrated by the Iranian Institute of Standards and Industrial Research [19].

Both blood and first-morning spot urine samples were obtained after overnight fasting, between 7:00 and 9:00 AM. Details on biochemical measurements [i.e., fasting serum glucose (FSG), 2-h serum glucose (2 h-SG), serum triglycerides (TG), total cholesterol, low density lipo-protein cholesterol (LDL-C), and high density lipo-protein cholesterol (HDL-C)] have been described elsewhere [20]. Urinary microalbumin concentration was measured using an ELISA kit (Padtan ELM Company, Tehran, Iran) and a microplate ELISA reader. Intra- and inter-assay coefficients of variations (CVs) were 9.3 and 9.8%, respectively. Urinary and serum Cr concentrations were determined by the Jaffe kinetic alkaline picrate method.

### Definition of terms

MetS were defined according to the NCEP ATP III diagnostic criteria [21]. For WC, we used the modified cutoff points for Iranian adults [22]. Having at least 3 following metabolic abnormalities was considered as MetS: 1-Hyperglycemia (FSG ≥ 100 mg/dL (5.6 mmol/L) or self-reported taking blood glucose-lowering medication); 2-Hypertriglyceridemia (serum TG ≥ 150 mg/dL (1.69 mmol/L) or using lipid-lowering drugs); 3-Low HDL-c (serum HDL-C < 40 mg/dL (1.04 mmol/L) for men, and < 50 mg/dL (1.29 mmol/L) for women or drug treatment); 4-Hypertension (blood pressure ≥ 130/85 mm Hg or drug treatment for hypertension), and 5-Abdominal obesity (WC≥ 95 cm for both genders). Different MetS phenotypes, represented by any three or more combinations of the five MetS components, were defined using the defined letters [23].

T2DM were defined as FSG ≥126 mg/dL, 2-hSG ≥200 mg/dL, or taking oral anti-diabetic medications [24]. Impaired fasting glucose (IFG) and impaired glucose tolerance (IGT) were defined as elevated FSG level (100–126 mg/dL) and 2 h-SG concentration (140–199 mg/dL), respectively [25].

Microalbuminuria was defined as urinary microalbumin concentrations of 20-200 mg/L [1]. The CKD-EPI creatinine equation, developed by Chronic Kidney Disease Epidemiology Collaboration, was used to calculate the estimated glomerular filtration rate (eGFR). As a single equation CKD-EPI has been expressed as follows: eGFR = 141 × min (S_cr_/κ, 1)^α^ × max (S_cr_/κ, 1)^-1.209^ × 0.993^age^ × 1.018 [if female] × 1.159 [if black]. In this equation, S_cr_ is serum Cr in mg/dL; κ is 0.7 and 0.9 for men and women, respectively; α is − 0.329, and − 0.411 for men and women, respectively, and min indicates the minimum of S_cr_/κ or 1, and max indicates the maximum of S_cr_/κ or 1 [26].

### Statistical analyses

Differences between general characteristics of the participants were compared across MetS and T2DM as dichotomous variables (yes/no), using independent sample t-test or chi-square test for quantitative and qualitative variables, respectively.

Multivariable logistic regression analyses were conducted to estimate the odds ratios (ORs) and 95% confidence intervals (CIs) of having T2DM, MetS, and MetS components across tertile categories of microalbumin concentrations and tertile categories of MCR. Potential confounding variables were entered into univariate models to determine confounders; those with P_E_ < 0.2 were considered potential confounders. Finally, confounders adjusted in the models were sex (male or female), age (year), BMI (kg/m^2^), and current smoking (yes/no).

The optimal cut-off points of urinary microalbumin and MCR for predicting the chance of having T2DM and MetS were determined using the receiver operator characteristic (ROC) curve analysis with an estimation of variables’ sensitivity and specificity. The cut-off points’ usefulness was assessed by the maximum value of sensitivity + specificity – 1 (Youden index); the index is preferable for finding the optimal cut-off points because it is clinically translated to maximizing correct classification and minimizing misclassification rates [27].

All statistical analyses were performed using the Statistical Package for Social Science (version 20; IBM Corp., Armonk, NY, USA) and MedCalc Statistical Software version 15.8 (MedCalc Software bvba, Ostend, Belgium). A *P*-value < 0.05 is considered significant.

## Results

The mean age (±SD) of participants was 44.9 ± 14.0 years, and 44.6% of the participants were men. The overall prevalence of microalbuminuria was 14.4%. Compared to controls, the prevalence of microalbuminuria was higher in the subjects with T2DM (19.4% vs. 12.8%) and MetS (16.7% vs. 12.8%). The general characteristics of the participants are shown in Table 1. Subjects with MetS or T2DM were more likely to be older, had higher values of BMI, WC, SBP and DBP, TG/HDL-C ratio, serum Cr, and urinary microalbumin, and lower levels of eGFR than those without MetS or T2DM. Furthermore, subjects with MetS were more likely to be men, and subjects with T2DM had lower urinary Cr and serum LDL-C concentration. Also, subjects with MetS or T2DM had significantly higher levels of MCR.

**Table 1:** Characteristics of the study population: Tehran Lipid and Glucose Study

Characteristic	MetS		T2DM	
	No (***n*** = 720)	Yes (***n*** = 438)	No (***n*** = 924)	Yes (***n*** = 139)
Age (y)	40.8 ± 12.9	52.1 ± 12.8*	44.3 ± 13.1	55.9 ± 11.7*
Female (%)	58.1%	47.5%*	54.4%	49.6%
Body mass index (kg/m^2^)	26.3 ± 4.56	30.4 ± 4.51*	27.8 ± 4.85	30.3 ± 5.10*
Waist circumference (cm)	89.3 ± 11.4	101 ± 9.54*	93.4 ± 11.8	101.1 ± 10.7*
SBP (mmHg)	108 ± 14.1	122 ± 16.1*	112 ± 15.7	123 ± 17.2*
DBP (mmHg)	73.8 ± 9.28	80.7 ± 9.54*	75.9 ± 10.1	79.8 ± 9.21*
TG/HDL ratio	2.35 ± 1.71	5.36 ± 4.34*	3.36 ± 3.00	4.89 ± 5.08*
Serum LDL-C (mg/dL)	110 ± 30.8	109 ± 35.7	111 ± 31.7	105 ± 39.1*
Serum creatinine (mg/dL)	1.07 ± 0.15	1.14 ± 0.17*	1.09 ± 0.16	1.15 ± 0.19*
eGFR (mL/min per 1.73m^2^)	75.6 ± 12.2	66.8 ± 13.2*	73.0 ± 12.5	64.3 ± 13.4*
Urinary microalbumin (mg/L)	14.1 ± 30.3	19.9 ± 45.9*	13.1 ± 25.8	35.3 ± 72.9*
Urinary creatinine (mg/L)	1580 ± 701	1537 ± 725	1587 ± 717	1417 ± 668*
Microalbumin to creatinine ratio (mg/mg)	0.011 ± 0.030	0.018 ± 0.057*	0.010 ± 0.026	0.036 ± 0.096*
Microalbuminuria (%)	12.8%	16.7%*	12.8%	19.4%*

*MetS* metabolic syndrome, *T2DM* type 2 diabetes mellitus, *SBP* systolic blood pressure, *DBP* diastolic blood pressure, *TG* triglyceride, *HDL-C* high density lipoprotein-cholesterol, *LDL-C* low density lipoprotein-cholesterol, *eGFR* estimated glomerular filtration rate

Values are expressed as mean ± SD

* *P* value < 0.05

**Fig. 1:**
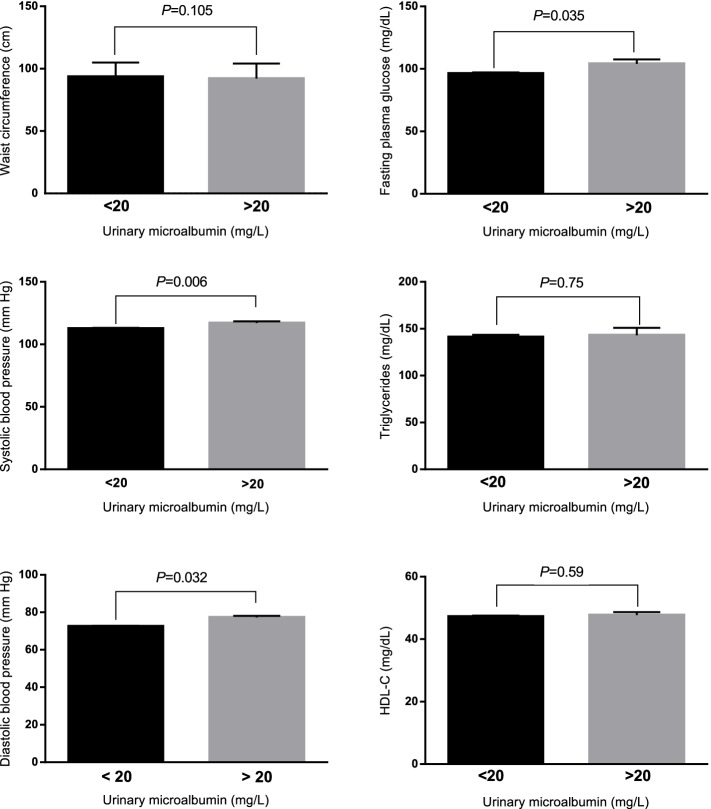
Mean (±SD) of cardio-metabolic variables in subjects with and without microalbuminuria

The mean (±SD) of cardio-metabolic variables in subjects with and without microalbuminuria are shown in Fig. 1. Subjects with microalbuminuria had higher SBP (116 ± 18.3 mmHg vs. 112 ± 15.9 mmHg), DBP (77.7 ± 11.4 mmHg vs. 76.0 ± 9.7 mmHg) and FSG (104 ± 46.2 mg/dL vs. 96.3 ± 26.6 mg/dL), compared to subjects with normal levels of microalbumin.

 ORs and 95% CIs of T2DM and dysglycemic conditions (i.e., IFG, IGT, IFG-IGT) across tertile categories of microalbumin concentration and MCR are shown in Table 2. After adjustment of confounding variables, subjects in the 3rd tertile of microalbumin concentration (> 19.4 mg/L) had a significantly higher chance of having T2DM than subjects in the first tertile, in either crude or adjusted models (OR = 2.01, 95% CI = 1.29-3.12, OR = 2.29, 95% CI = 1.43-3.67, respectively). Also, the adjusted chance of having T2DM was significantly elevated in the highest category of MCR (OR = 1.82, 95% CI = 1.15-2.89). Neither IFG nor IGT, and their combination, had no significant association with urinary microalbumin concentrations or MCR.

**Table 2:** Odds ratio and 95% CI of type 2 diabetes and dysglycemic conditions across tertiles of microalbumin and microalbumin-to-creatinine ratio

	Microalbumin		MCR		Microalbuminuria^a^
	Tertile 2	Tertile 3	Tertile 2	Tertile 3	
**Diabetes Mellitus**					
Crude	1.09 (0.68-1.76)	2.01 (1.29-3.12)*	0.88 (0.54-1.42)	1.89 (1.23-2.89)*	1.65 (1.04-2.61)
Model 1	1.23 (0.74-2.04)	2.29 (1.43-3.67)*	0.92 (0.55-1.52)	1.82 (1.15-2.89)*	1.75 (1.06-2.88)
**IFG**					
Crude	1.66 (0.99-2.80)	1.13 (0.65-1.97)	0.90 (0.53-1.51)	1.00 (0.60-1.67)	0.70 (0.35-1.37)
Model 1	1.73 (1.02-2.94)	1.24 (0.71-2.19)	0.94 (0.55-1.59)	1.06 (0.63-1.78)	0.74 (0.37-1.46)
**IGT**					
Crude	1.04 (0.58-1.86)	1.18 (0.67-2.07)	0.60 (0.33-1.11)	1.01 (0.59-1.73)	1.35 (0.74-2.46)
Model 1	1.05 (0.58-1.89)	1.18 (0.66-2.11)	0.59 (0.32-1.09)	0.97 (0.56-1.67)	1.28 (0.68-2.41)
**IFG/IGT**					
Crude	1.00 (0.51-1.95)	1.00 (0.51-1.95)	0.62 (0.30-1.30)	1.24 (0.66-2.31)	1.55 (0.78-3.07)
Model 1	1.14 (0.57-2.27)	1.11 (0.56-2.23)	0.67 (0.31-1.43)	1.23 (0.64-2.35)	1.70 (0.83-3.46)

*MCR* microalbumin-to-creatinine ratio, *IFG* impaired fasting glucose, *IGT* impaired glucose tolerance

Model 1 adjusted for sex (male/female), age (years), BMI (kg/m^2^), current smoking (yes/no)

Median of microalbumin concentrations in the first, second and third tertiles were 2.14, 8.07, and 19.4 mg/L, respectively

Median of MCR in the first, second and third tertiles were 0.0015, 0.0052, and 0.015 mg/mg, respectively

^a^ Odds (and 95% CIs) in subjects with microalbuminuria (urinary microalbumin concentration 20-200 mg/L) compared to subjects with normal values (urinary microalbumin concentration < 20 mg/L)

* *P* for trend < 0.05

ORs with 95% CIs of MetS and its components across tertile categories of microalbumin concentration and tertile categories of MCR are shown in Table 3. After adjustment of confounding variables, subjects in the 3rd tertile of microalbumin concentration (> 19.4 mg/L) had a significantly higher chance of having MetS than subjects in the first tertile (OR = 1.66, 95% CI = 1.17-2.35). Compared to the first tertile (< 2.14 mg/L), subjects in the 3rd tertile of urinary microalbumin had 63 and 78% increased chance of having hypertension (OR = 1.63, 95% CI = 1.16-2.30) and hyperglycemia (OR = 1.78, 95% CI = 1.24-2.56). Moreover, subjects in the 3rd tertile of MCR had significantly higher odds of hypertension (OR = 1.55, 95% CI = 1.10-2.18) and hyperglycemia (OR = 1.45, 95% CI = 1.02-2.06). However, there was no significant association between microalbumin level or MCR and other components of MetS.

**Table 3:** Odds ratios and 95% CIs of the MetS and its components across tertiles of urinary microalbumin and microalbumin-to-creatinine ratio

	Microalbumin		MCR		Microalbuminuria^a^
	Tertile 2	Tertile 3	Tertile 2	Tertile 3	
**Metabolic syndrome**					
Crude	0.91 (0.68-1.22)	1.26 (0.94-1.68)	0.81 (0.61-1.09)	1.22 (0.92-1.63)	1.36 (0.98-1.90)
Adjusted	0.96 (0.68-1.35)	1.66 (1.17-2.35)*	0.77 (0.54-1.08)	1.28 (0.91-0.81)	1.65 (1.10-2.48)
**Abdominal obesity**					
Crude	1.04 (0.77-1.39)	0.78 (0.58-1.03)	0.88 (0.66-1.18)	0.84 (0.63-1.12)	0.99 (0.71-1.39)
Adjusted	1.24 (0.78-1.98)	0.99 (0.60-1.62)	0.94 (0.59-1.49)	1.01 (0.61-1.66)	1.52 (0.81-2.85)
**Hypertension**					
Crude	0.71 (0.52-0.98)	1.31 (0.94-1.77)	0.81 (0.59-1.11)	1.48 (1.09-1.99)*	1.80 (1.29-2.52)
Adjusted	0.69 (0.49-0.99)	1.63 (1.16-2.30)*	0.77 (0.54-1.09)	1.55 (1.10-2.18)*	2.37 (1.59-3.51)
**Hyperglycemia**					
Crude	1.21 (0.87-1.69)	1.44 (1.04-1.99)*	0.81 (0.58-1.13)	1.42 (1.03-1.94)*	1.27 (0.89-1.82)
Adjusted	1.37 (0.96-1.97)	1.78 (1.24-2.56)*	0.81 (0.56-1.16)	1.45 (1.02-2.06)*	1.45 (0.97-2.18)
**Hypertriglyceridemia**					
Crude	1.06 (0.80-1.41)	1.06 (0.80-1.41)	0.82 (0.62-1.09)	1.05 (0.79-1.40)	0.98 (0.71-1.37)
Adjusted	1.12 (0.82-1.53)	1.27 (0.92-1.75)	0.81 (0.59-1.11)	1.11 (0.81-1.53)	1.05 (0.72-1.53)
**Low HDL-C**					
Crude	0.91 (0.69-1.21)	1.11 (0.84-1.47)	0.92 (0.69-1.22)	1.14 (0.86-1.52)	1.19 (0.86-1.65)
Adjusted	0.95 (0.71-1.27)	1.20 (0.89-1.61)	0.93 (0.70-1.25)	1.14 (0.85-1.53)	1.28 (0.91-1.81)

*MCR* microalbumin-to-creatinine ratio, *HDL-C* high density lipoprotein cholesterol

Adjusted for sex (male/female), age (years), BMI (kg/m^2^), current smoking (yes/no)

Median of microalbumin concentrations in the first, second and third tertiles were 2.14, 8.07, and 19.4 mg/L, respectively

Median of MCR in the first, second and third tertiles were 0.0015, 0.0052, and 0.015 mg/mg, respectively

^a^ Odds (and 95% CI) in subjects with microalbuminuria (urinary microalbumin concentration 20-200 mg/L) compared to subjects with normal values (urinary microalbumin concentration < 20 mg/L)

* *P* for trend < 0.05

ORs with 95% CIs of different MetS phenotypes across tertiles of urinary microalbumin are shown in Supplementary Table 1. Among 15 different phenotypes, WGT (elevated WC, elevated blood glucose, elevated TG), WGB (elevated WC, elevated blood glucose, elevated blood pressure), and WBH (elevated WC, elevated blood pressure, low HDL-C) had significant associations with urinary microalbumin concentration (OR = 1.57, 95% CI = 1.03-2.41 for WGT, OR = 2.00, 95% CI = 1.24-3.24 for WGB, OR = 1.52, 95% CI = 1.01-2.30 for WBH).

After adjustment for confounding variables, subjects with microalbuminuria had increased chance of having T2DM (OR = 1.75, 95% CI = 1.06-2.88) (Table 2), MetS (OR = 1.65, 95% CI = 1.10-2.48) and hypertension (OR = 2.37, 95% CI = 1.59-3.51) (Table 3). Each 10 mg/L increased urinary microalbumin concentration was related to increased odds of MetS and T2DM by 5% (OR = 1.05, 95% CI = 1.01-1.10) and 10% (OR = 1.10, 95% CI = 1.06-1.15), respectively.

**Fig. 2:**
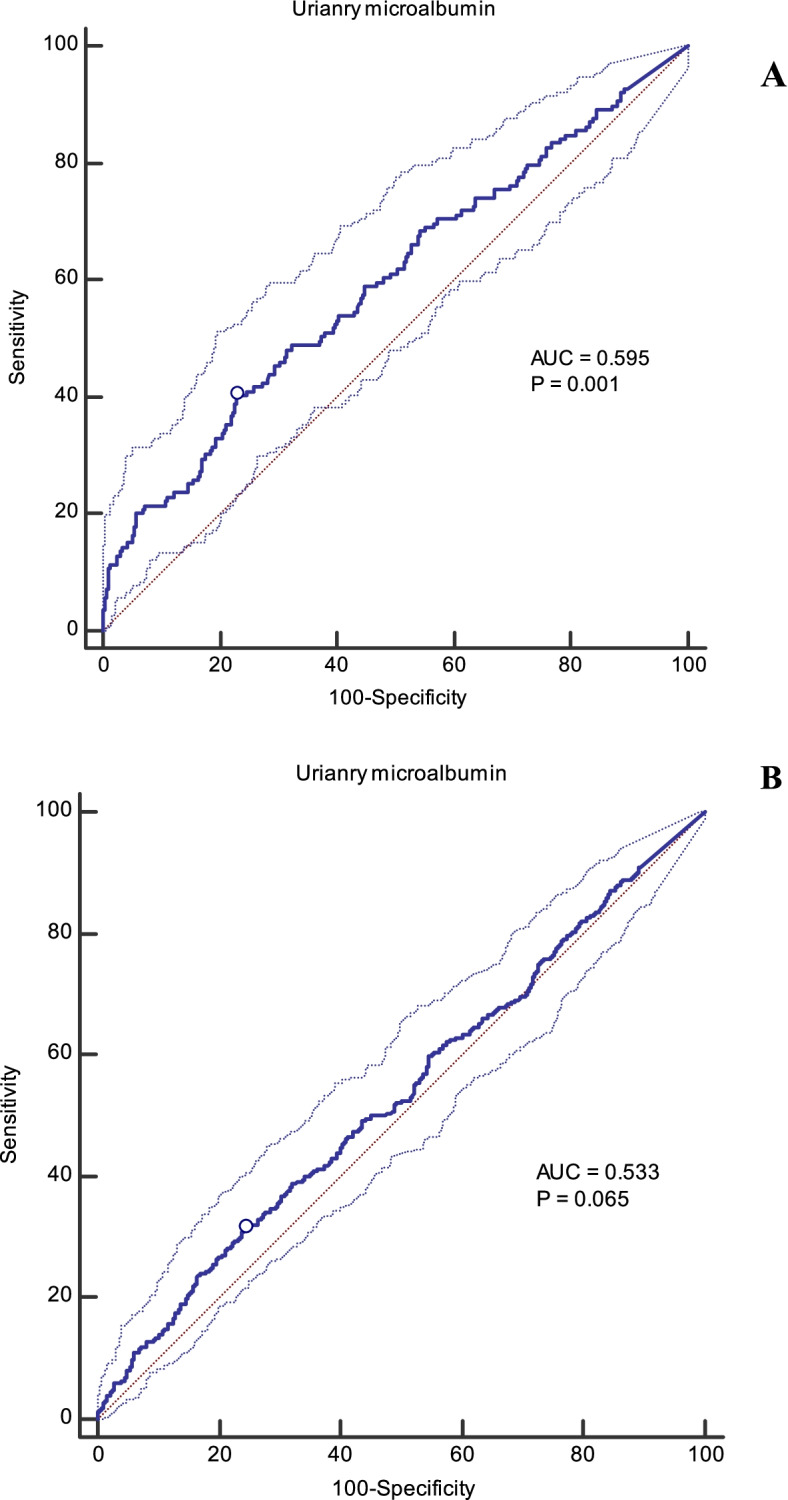
**A** Cut-off point of urinary microalbumin for chance of having T2DM (14.0 mg/L, sensitivity = 40.3, specificity = 77.1, Youden index = 0.17). **B**. Cut-off point of urinary microalbumin for chance of having MetS (13.6 mg/L, sensitivity = 31.5, specificity = 75.7, Youden index = 0.07)

**Fig. 3:**
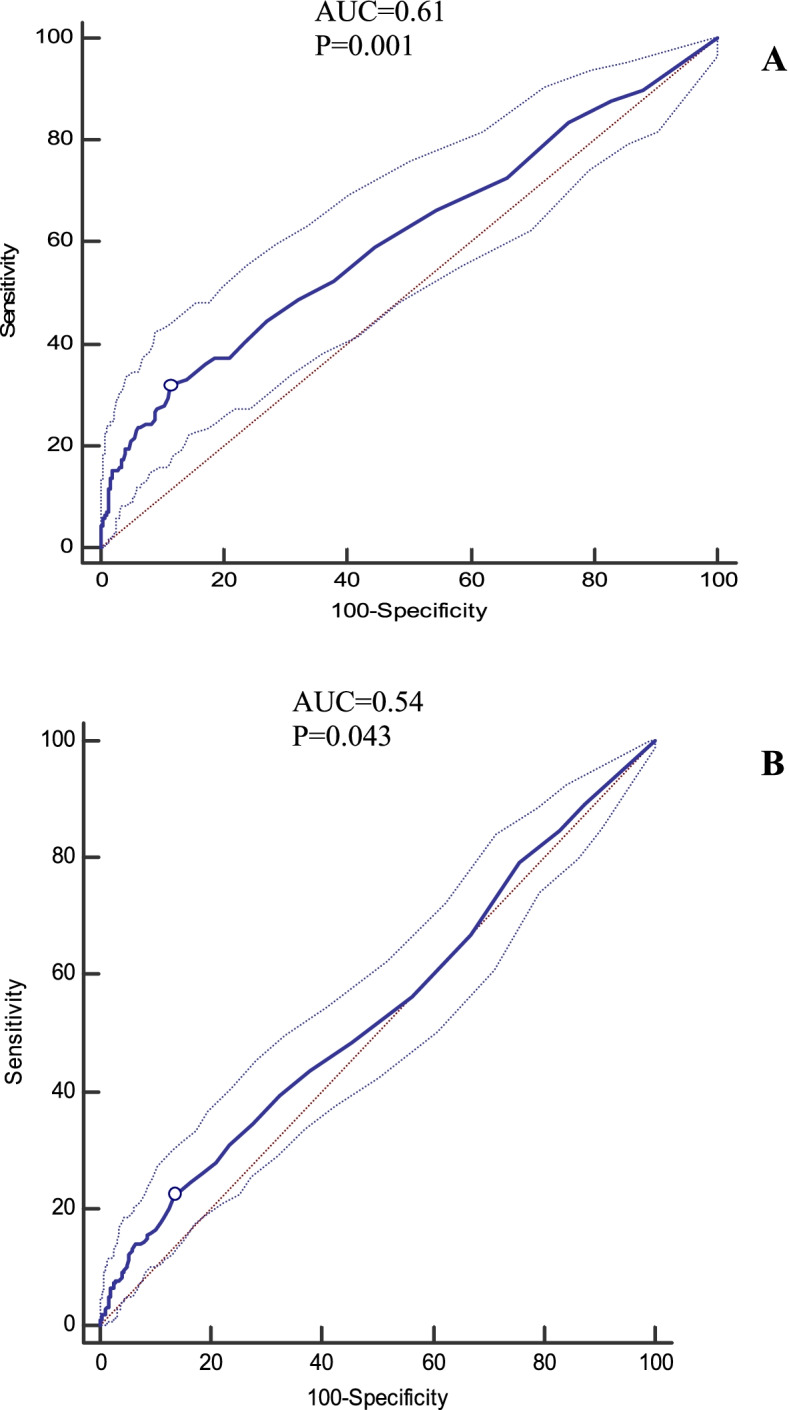
**A** Cut-off point of urinary microalbumin-to-creatinine (MCR) for chance of having T2DM (0.016, sensitivity = 32.4, specificity =87.8, Youden index = 0.20). **B**. Cut-off point of urinary MCR for chance of having MetS (0.014, sensitivity = 22.4, specificity =86.4, Youden index = 0.09)

Optimal cut-off values of urinary microalbumin and MCR for the chance of having T2DM and MetS, as well as sensitivity, specificity, and AUC (*P*-value), are presented in Figs. 2 and 3. The optimal cut-off points of urinary microalbumin levels for the chance of having T2DM and MetS were 14.0 and 13.6 mg/L, respectively. The optimal cut-off points of MCR for the chance of having T2DM and MetS were 0.016 and 0.014 mg/mg, respectively.

## Discussion

In this population-based study, we found that individuals with elevated urinary microalbumin concentrations had a significantly higher chance of having MetS and T2DM. Moreover, there were positive associations between microalbumin concentrations and some components of MetS, including hypertension and hyperglycemia. Each 10 mg/L increased urinary microalbumin concentration was related to increased odds of MetS and T2DM by 5 and 10%, respectively. The optimal cut-off values of urinary microalbumin for predicting the chance of having T2DM and MetS were determined as 14.0 and 13.6 mg/L, respectively. These values were lower than those generally considered as microalbuminuria. To the best of our knowledge, the association between spot urinary microalbumin concentration and the chance of having MetS and T2DM has not been investigated among the Iranian population. The optimal cut-off values of urinary microalbumin and MCR for predicting the chance of having T2DM and MetS have also not yet been determined.

Findings of this population-based study indicated strong associations between urinary microalbumin and metabolic disorders. These findings also provided further evidence to the emerging literature suggesting elevated urinary microalbumin level as a prognostic surrogate of cardio-metabolic outcomes. Current cut-off levels, considered the threshold of microalbuminuria, are debatable [28]. The levels of micro-albumin, below the values usually defined as microalbuminuria, could strongly predict risk of cardio-metabolic complications [29].

A cross-sectional study of 8497 Korean adults showed a significant association between MetS and risk of microalbuminuria, with about 3-fold increased risk in subjects with MetS compared to healthy subjects [30]; all components of the MetS especially hypertension, were associated with increased risk of microalbuminuria [30]. The NHANES study reported a significant association between microalbuminuria and the risk of hypertension and hyperglycemia in men and women [9]. In an 11-year follow-up of 882 adults aged 20-74 years, the risk of developing T2DM was reported as 1.90 (95% CI = 0.88-4.06) and 2.51 (95% CI = 1.08-5.87) for those with microalbuminuria and macro-albuminuria, respectively [31]. The prevalence of microalbuminuria in diabetic patients is approximately 20-40% in different populations [32–34], that could be considered an early sign of diabetic nephropathy and other diabetic complications. These indicate the need for appropriate screening programs and strict control of modifiable risk factors to reduce future complications. On the other hand, in the present study we observed that T2DM patients had a significant lower level of LDL-C compared to those without T2DM, the finding was likely due to the medication with lipid lowering drug in T2DM patients.

Also, the prevalence of microalbuminuria in the groups of MetS patients was reported at 20.3% in China [10], 20.8% in Japan [11], and 5.4% in the Korean population [6], which may indicate the need for early screening strategies for prevention and treatment of microalbuminuria in patients with MetS, and also early management of MetS and its complications to prevent the progression of renal injury. The discrepancy in the prevalence of microalbuminuria across these populations might be attributable to the differences in the criteria defined for microalbuminuria or differences in the characteristics of study participants, such as age and cardiovascular risk factors.

The underlying mechanisms connecting urinary microalbumin levels with developing metabolic disorders are not fully established. Microalbuminuria is mainly accompanied by vascular damage and endothelial dysfunction [35], resulting in MetS and T2DM [36]. On the other hand, the presence of MetS components such as abdominal obesity, hypertension, or hyperglycemia could be responsible for increased endothelial permeability and intra-glomerular capillary pressure, resulting in kidney dysfunction and microalbuminuria [14].

The strengths of our study include the relatively large and representative sample and defining comprehensive models to adjust the significant potential confounders. However, the present study has some limitations. First, it was a cross-sectional study, and we cannot conclude any causal inference between urinary microalbumin concentrations and the risk of developing MetS and T2DM. Well-designed prospective cohort studies with a long follow-up period should conduct to confirm the association between spot urinary microalbumin and risk of MetS and T2DM incidence. Although previous studies indicated an association between elevated urinary microalbumin and metabolic disorders, the current study addressed new cut-off values (both for urinary microalbumin and MCR) for predicting T2DM and MetS. However, prospective studies are needed to examine the predictive power of the cut-offs for the development and prognosis of the outcomes. Second, urinary microalbumin was determined based on spot urine samples, which may lead to ignoring day-to-day differences in the urinary microalbumin concentration and potential differences at different hours of a day. Although the spot urine sampling is cost-effective and straightforward for population-based studies and has some advantages, i.e., rapid, deep freezing after collection in the absence of any antiseptic agent, it might be less accurate than the 24-h urine collections for the assessment of albumin excretion. However, studies reported a good correlation between spot urine, either first morning or random morning urine, with the 24-h albumin excretion rate [37]. Third, the ROC curve analyses indicated a relatively small effect size of microalbumin for predicting T2DM and MetS in the general population; however, a small effect size epidemiologic studies or large-scale assessment may be translated into a relevant magnitude in clinical settings. In turn, such population-based cut-off estimation may be favorably used to promote diagnostic criteria in clinical practices.

## Conclusions

In conclusion, elevated urinary microalbumin concentrations even below the cut-off determined as microalbuminuria was associated with the chance of having T2DM and MetS. However, prospective well-designed cohorts are needed to confirm the spot urinary microalbumin measurement as a strong predictor of cardiometabolic events.

## Supplementary Information

**Additional file 1: Supplementary Table 1.** Odds ratios and 95% CIs of different combinations of MetS components across tertiles of urinary microalbumin concentration.

## Data Availability

The datasets used and/or analyzed during the current study available from the corresponding author on reasonable request.

## References

[1] Lambers Heerspink HJ, Brantsma AH, de Zeeuw D, Bakker SJ, de Jong PE, Gansevoort RT (2008). Albuminuria assessed from first-morning-void urine samples versus 24-hour urine collections as a predictor of cardiovascular morbidity and mortality. Am J Epidemiol.

[2] Gerstein HC, Mann JF, Yi Q, Zinman B, Dinneen SF, Hoogwerf B (2001). Albuminuria and risk of cardiovascular events, death, and heart failure in diabetic and nondiabetic individuals. JAMA.

[3] Li XH, Lin HY, Wang SH, Guan LY, Wang YB (2016). Association of Microalbuminuria with metabolic syndrome among aged population. Biomed Res Int.

[4] Pan CY, Ho LT, Soegondo S, Prodjosudjadi W, Suwanwalaikorn S, Lim SC (2008). Prevalence of albuminuria and cardiovascular risk profile in a referred cohort of patients with type 2 diabetes: an Asian perspective. Diabetes Technol Ther.

[5] Sheng C-S, Hu B-C, Fan W-X, Zou J, Li Y, Wang J-G (2011). Microalbuminuria in relation to the metabolic syndrome and its components in a Chinese population. Diabetol Metab Syndr.

[6] Kim YS, Kim HS, Oh HY, Lee MK, Kim CH, Kim YS (2013). Prevalence of microalbuminuria and associated risk factors among adult Korean hypertensive patients in a primary care setting. Hypertens Res.

[7] Pontremoli R, Sofia A, Ravera M, Nicolella C, Viazzi F, Tirotta A (1997). Prevalence and clinical correlates of microalbuminuria in essential hypertension: the MAGIC Study. Microalbuminuria: A Genoa Investigation on Complications. Hypertension (Dallas, Tex : 1979).

[8] Tanaka S, Takase H, Dohi Y, Kimura G (2013). The prevalence and characteristics of microalbuminuria in the general population: a cross-sectional study. BMC Res Notes.

[9] Palaniappan L, Carnethon M, Fortmann SP (2003). Association between microalbuminuria and the metabolic syndrome: NHANES III. Am J Hypertens.

[10] Chen B, Yang D, Chen Y, Xu W, Ye B, Ni Z (2010). The prevalence of microalbuminuria and its relationships with the components of metabolic syndrome in the general population of China. Clin Chim Acta.

[11] Hao Z, Konta T, Takasaki S, Abiko H, Ishikawa M, Takahashi T (2007). The association between microalbuminuria and metabolic syndrome in the general population in Japan: the Takahata study. Int Med (Tokyo, Japan).

[12] Afkhami-Ardekani M, Modarresi M, Amirchaghmaghi E (2008). Prevalence of microalbuminuria and its risk factors in type 2 diabetic patients. Indian J Nephrol.

[13] Go RCP, Desmond R, Roseman JM, Bell DSH, Vanichanan C, Acton RT (2001). Prevalence and risk factors of microalbuminuria in a cohort of African-American women with gestational diabetes. Diabetes Care.

[14] Bonnet F, Marre M, Halimi JM, Stengel B, Lange C, Laville M (2006). Waist circumference and the metabolic syndrome predict the development of elevated albuminuria in non-diabetic subjects: the DESIR study. J Hypertens.

[15] Lee S-H, Kim DH, Kim Y-H, Roh YK, Ju SY, Nam H-Y (2016). Relationship Between Dyslipidemia and Albuminuria in Hypertensive Adults: A Nationwide Population-Based Study. Medicine (Baltimore).

[16] Li Q, Jia WP, Lu JQ, Chen L, Wu YM, Jiang SY (2004). Relationship between the prevalence of microalbuminuria and components of metabolic syndrome in Shanghai. Zhonghua Liu Xing Bing Xue Za Zhi.

[17] Lin CC, Liu CS, Li TC, Chen CC, Li CI, Lin WY (2007). Microalbuminuria and the metabolic syndrome and its components in the Chinese population. Eur J Clin Investig.

[18] Azizi F, Zadeh-Vakili A, Takyar M (2018). Review of Rationale, Design, and Initial Findings: Tehran Lipid and Glucose Study. Int J Endocrinol Metab.

[19] Askari S, Asghari G, Ghanbarian A, Khazan M, Alamdari S, Azizi F (2014). Seasonal variations of blood pressure in adults: Tehran lipid and glucose study. Arch Iranian Med.

[20] Tohidi M, Ghasemi A, Hadaegh F, Derakhshan A, Chary A, Azizi F (2014). Age- and sex-specific reference values for fasting serum insulin levels and insulin resistance/sensitivity indices in healthy Iranian adults: Tehran lipid and glucose study. Clin Biochem.

[21] Grundy SM, Cleeman JI, Daniels SR, Donato KA, Eckel RH, Franklin BA (2005). Diagnosis and management of the metabolic syndrome: an American Heart Association/National Heart, Lung, and Blood Institute scientific statement. Circulation.

[22] Azizi F, Hadaegh F, Khalili D, Esteghamati A, Hosseinpanah F, Delavari A (2010). Appropriate definition of metabolic syndrome among Iranian adults: report of the Iranian National Committee of obesity. Arch Iranian Med.

[23] Mirmiran P, Bahadoran Z, Tahmasebinejad Z, Azizi F, Ghasemi A (2019). Circulating nitric oxide metabolites and the risk of cardiometabolic outcomes: a prospective population-based study. Biomarkers.

[24] American DA (2011). Diagnosis and classification of diabetes mellitus. Diabetes Care.

[25] Report of the Expert Committee on the Diagnosis and Classification of Diabetes Mellitus. Diabetes care. 2003;26(suppl 1):s5-s20. 10.2337/diacare.26.2007.s5 12502614

[26] Levey AS, Stevens LA, Schmid CH, Zhang YL, Castro AF, Feldman HI (2009). A new equation to estimate glomerular filtration rate. Ann Intern Med.

[27] Perkins NJ, Schisterman EF (2006). The inconsistency of "optimal" cutpoints obtained using two criteria based on the receiver operating characteristic curve. Am J Epidemiol.

[28] Ellam TJ, El Nahas M (2011). Proteinuria thresholds are irrational: a call for proteinuria indexing. Nephron Clin Pract.

[29] Ibsen H, Olsen MH, Wachtell K, Borch-Johnsen K, Lindholm LH, Mogensen CE (2008). Reduction in albuminuria translates to reduction in cardiovascular events in hypertensive patients with left ventricular hypertrophy and diabetes. J Nephrol.

[30] Lee HO, Bak HJ, Shin JY, Song YM (2015). Association between metabolic syndrome and microalbuminuria in Korean adults. Korean J Fam Med.

[31] Wang Z, Hoy WE (2006). Albuminuria as a marker of the risk of developing type 2 diabetes in non-diabetic Aboriginal Australians. Int J Epidemiol.

[32] Ahmad T, Ulhaq I, Mawani M, Islam N (2017). Microalbuminuria in Type-2 diabetes mellitus; the tip of iceberg of diabetic complications. Pak J Med Sci.

[33] Pasko N, Toti F, Strakosha A, Thengjilli E, Shehu A, Dedej T (2013). Prevalence of microalbuminuria and risk factor analysis in type 2 diabetes patients in Albania: the need for accurate and early diagnosis of diabetic nephropathy. Hippokratia.

[34] Thakur SK, Dhakal SP, Parajuli S, Sah AK, Nepal SP, Paudel BD (2019). Microalbuminuria and its risk factors in type 2 diabetic patients. J Nepal Health Res Counc.

[35] Ochodnicky P, Henning RH, van Dokkum RP, de Zeeuw D (2006). Microalbuminuria and endothelial dysfunction: emerging targets for primary prevention of end-organ damage. J Cardiovasc Pharmacol.

[36] Levy BI, Schiffrin EL, Mourad JJ, Agostini D, Vicaut E, Safar ME (2008). Impaired tissue perfusion: a pathology common to hypertension, obesity, and diabetes mellitus. Circulation.

[37] Soonthornpun S, Leelawattana R, Thamprasit A, Rattarasarn C, Setasuban W, Thammakumpee N (2002). Screening for microalbuminuria in type 2 diabetes: a reconsideration. J Med Assoc Thai.

