# Supplementary material for: Spot urinary microalbumin concentration, metabolic syndrome and type 2 diabetes: Tehran lipid and glucose study
Source: BMC Endocr Disord. 2022 Mar 8;22:59. doi: 10.1186/s12902-022-00976-x (PMC8905801; doi:10.1186/s12902-022-00976-x)
Supplement: Supplementary file 1 — Additional file 1: Supplementary Table 1. Odds ratios and 95% CIs of different combinations of MetS components across tertiles of urinary microalbumin concentration. [file 12902_2022_976_MOESM1_ESM.docx]

**Supplementary Table 1.** Odds ratios and 95% CIs of different combinations of MetS components across tertiles of urinary microalbumin concentration

| **Micro-albumin** | **Tertile 1**  **(n=397)** | **Tertile 2**  **(n=399)** | **Tertile 3**  **(n=396)** | **P for trend** |
| --- | --- | --- | --- | --- |
| WGH |  |  |  |  |
| Crude | 1.00 | 1.00 (0.67-1.48) | 1.14 (0.78-1.68) | 0.489 |
| Adjusted | 1.00 | 1.15 (0.75-1.78) | 1.37 (0.89-2.10) | 0.152 |
| WGT |  |  |  |  |
| Crude | 1.00 | 1.02 (0.68-1.52) | 1.30 (0.89-1.91) | 0.169 |
| Adjusted | 1.00 | 1.16 (0.75-1.79) | 1.57 (1.03-2.41) | 0.037 |
| WGB |  |  |  |  |
| Crude | 1.00 | 1.00 (0.63-1.58) | 1.59 (1.04-2.43) | 0.028 |
| Adjusted | 1.00 | 1.18 (0.71-1.96) | 2.00 (1.24-3.24) | 0.004 |
| WBH |  |  |  |  |
| Crude | 1.00 | 0.70 (0.46-1.04) | 1.25 (0.87-1.80) | 0.206 |
| Adjusted | 1.00 | 0.74 (0.47-1.15) | 1.52 (1.01-2.30) | 0.047 |
| WBT |  |  |  |  |
| Crude | 1.00 | 0.75 (0.51-1.12) | 1.19 (0.82-1.71) | 0.337 |
| Adjusted | 1.00 | 0.79 (0.51-1.22) | 1.41 (0.94-2.13) | 0.101 |
| BHT |  |  |  |  |
| Crude | 1.00 | 0.71 (0.47-1.06) | 1.24 (0.85-1.79) | 0.237 |
| Adjusted | 1.00 | 0.74 (0.47-1.15) | 1.42 (0.94-2.15) | 0.092 |
| BGT |  |  |  |  |
| Crude | 1.00 | 0.86 (0.53-1.39) | 1.32 (0.85-1.06) | 0.200 |
| Adjusted | 1.00 | 0.96 (0.57-1.61) | 1.52 (0.94-2.48) | 0.086 |
| BGH |  |  |  |  |
| Crude | 1.00 | 0.85 (0.52-1.40) | 1.34 (0.85-2.11) | 0.190 |
| Adjusted | 1.00 | 0.98 (0.57-1.67) | 1.56 (0.94-2.56) | 0.079 |
| WHT |  |  |  |  |
| Crude | 1.00 | 0.78 (0.57-1.80) | 0.96 (0.70-1.32) | 0.806 |
| Adjusted | 1.00 | 0.80 (0.56-1.15) | 1.13 (0.79-1.61) | 0.527 |
| GHT |  |  |  |  |
| Crude | 1.00 | 0.93 (0.62-1.40) | 1.19 (0.81-1.77) | 0.363 |
| Adjusted | 1.00 | 1.04 (0.67-1.61) | 1.37 (0.89-2.01) | 0.150 |
| WGHB |  |  |  |  |
| Crude | 1.00 | 0.84 (0.50-1.41) | 1.30 (0.81-2.08) | 0.263 |
| Adjusted | 1.00 | 0.98 (0.56-1.72) | 1.53 (0.91-2.59) | 0.105 |
| WGHT |  |  |  |  |
| Crude | 1.00 | 0.91 (0.59-1.39) | 1.16 (0.77-1.74) | 0.462 |
| Adjusted | 1.00 | 1.02 (0.65-1.62) | 1.36 (0.87-2.12) | 0.181 |
| TGHB |  |  |  |  |
| Crude | 1.00 | 0.84 (0.51-1.40) | 1.26 (0.79-2.01) | 0.323 |
| Adjusted | 1.00 | 0.96 (0.56-1.66) | 1.43 (0.86-2.39) | 0.166 |
| WBGT |  |  |  |  |
| Crude | 1.00 | 0.79 (0.48-1.31) | 1.27 (0.81-2.01) | 0.276 |
| Adjusted | 1.00 | 0.89 (0.52-1.54) | 1.49 (0.90-2.46) | 0.117 |
| WBHT |  |  |  |  |
| Crude | 1.00 | 0.91 (0.59-1.39) | 1.16 (0.77-1.74) | 0.462 |
| Adjusted | 1.00 | 1.02 (0.65-1.62) | 1.36 (0.87-2.12) | 0.181 |

W, elevated waist circumference; G, elevated blood glucose; T, elevated triglyceride; H, low HDL-C; B, elevated blood pressure.

Adjusted for sex (male/female), age (years), BMI (kg/m^2^), current smoking (yes/no).

Median of micro-albumin concentrations in the first, second and third tertiles were 2.14, 8.07, and 19.4 mg/L, respectively.
